# Aging-Related Dissociation of Spatial and Temporal N400 in Sentence-Level Semantic Processing: Evidence From Source Analyses

**DOI:** 10.3389/fnagi.2022.877235

**Published:** 2022-06-10

**Authors:** Sora An, Se Jin Oh, Sang Beom Jun, Jee Eun Sung

**Affiliations:** ^1^Department of Communication Disorders, Ewha Womans University, Seoul, South Korea; ^2^Department of Electronic and Electrical Engineering, Ewha Womans University, Seoul, South Korea; ^3^Graduate Program in Smart Factory, Ewha Womans University, Seoul, South Korea; ^4^Department of Brain and Cognitive Sciences, Ewha Womans University, Seoul, South Korea

**Keywords:** aging, sentence processing, semantic processing, event-related potential, source analysis, brain network

## Abstract

Age-related differences in sentence-level lexical-semantic processes have been extensively studied, based on the N400 component of event-related potential (ERP). However, there is still a lack of understanding in this regard at the brain-region level. This study explores aging effects on sentence-level semantic processing by comparing the characteristics of the N400 ERP component and brain engagement patterns within individual N400 time windows for two age groups (16 younger adults aged 24.38 ± 3.88 years and 15 older adults aged 67.00 ± 5.04 years) during sentence processing with different plausibility conditions. Our results demonstrated that the N400 effect according to the plausibility condition occurred in different temporal windows in the two age groups, with a delay in the older group. Moreover, it was identified that there was a distinct difference between the groups in terms of the source location of the condition-dependent N400 effect even though no significant difference was derived in its magnitude itself at the sensor-level. Interestingly, the source analysis results indicated that the two groups involved different functional networks to resolve the same semantic violations: the younger group activated the regions corresponding to the typical lexical-semantic network more, whereas the older group recruited the regions belonging to the multiple-demand network more. The findings of this study could be used as a basis for understanding the aging brain in a linguistic context.

## Introduction

Although it is well known that linguistic-cognitive difficulties and burdens increase as people age, identifying the specific locus of impairment in finite linguistic levels associated with aging has been somewhat controversial (Abrams and Farrell, 2011). Previous studies have reported that older adults demonstrate greater difficulties in understanding sentences than younger adults due to cognitive decline, particularly involving reduced working memory capacity (Caplan and Waters, 2005; Caplan et al., 2011; DeDe and Flax, 2016). However, several studies argue that while older adults may encounter comprehension impairments, their real-time sentence-processing abilities are preserved until the later stage of aging (Kemtes and Kemper, 1997; Caplan and Waters, 2005; Thornton and Light, 2006). One of the most widely used methods for examining real-time sentence-processing in terms of the brain network system is the language-related event-related potential (ERP) paradigm. This is a method of analyzing the effect of specific sensory or cognitive events using time-locked average signals for the trigger event based on EEG signals that are recorded while sentences or phrases are presented as visual or auditory stimuli (Kutas and Federmeier, 2012).

To date, numerous studies analyzing aging-related effects in sentence-processing by applying the ERP paradigm have mainly focused on lexical-semantic processes based on the N400 component (Kutas and Federmeier, 2011; Wlotko and Federmeier, 2012; Tiedt et al., 2020). The N400 is a component of ERPs that exhibits a relatively negative-going potential compared to a specific reference electrode, which peaks around 400 ms post-stimulus onset and is observed around the central and parietal electrode positions (Tiedt et al., 2020). Conventionally, the N400 has been employed as a potential biomarker to identify lexical-semantic processing (Hajra et al., 2018) with two common indices: peak amplitude and peak latency.

According to more than 30 years of scientific debate, the peak amplitude of N400 reflects the amount of cognitive processing, encompassing access to long-term memory for the retrieval of relevant knowledge and semantic integration (Tiedt et al., 2020). Therefore, it tends to increase when the features of a word or sentence are not congruent with its context; this is referred to as the N400 effect (Kutas and Federmeier, 2000, 2011; Tiedt et al., 2020). Previous studies have reliably shown a reduced N400 effect among older adults compared to younger adults (Federmeier and Kutas, 2005; Federmeier et al., 2010; Payne and Federmeier, 2017) in relation to aging effects on the N400 in semantic processing within a sentential context. Researchers have suggested that this age-related amplitude reduction is attributed to normal aging processes, such as neuroanatomical changes leading to a decreased synchronous firing of neurons (Walhovd et al., 2005; Kemmotsu et al., 2012) or a decline in cognitive resources (Kramer and Kray, 2006; Chapman et al., 2007).

Latency prolongation has also been reported consistently in aging-related N400 studies: the peak latency among older adults tends to be delayed compared to younger adults (Kutas and Iragui, 1998; Federmeier and Kutas, 2005; Wlotko et al., 2010). Researchers have associated these delayed features with slowed information processing (Salthouse, 2000) or less-efficient processing and integration at the higher-order linguistic level (Iragui et al., 1996; Federmeier et al., 2003). In particular, Federmerier et al. have demonstrated that such age-dependent latency changes do not occur monotonically due to delays in the processing phase of sensory input, but can be induced by delays in the linguistic processing phase itself (Federmeier et al., 2003).

As described above, aging effects on the N400 component in the context of lexical-semantic processes have been extensively studied at the EEG sensor-level, identifying characteristic differences between younger and older adults (Kutas and Iragui, 1998; Federmeier and Kutas, 2005; Federmeier et al., 2010; Wlotko et al., 2010; Payne and Federmeier, 2017). Nevertheless, understanding at the brain region-level is still lacking in this regard. Several source-localization studies have reported that the most prominent source of the N400 is the temporal cortex, especially in the left hemisphere (Van Petten and Luka, 2006; Khateb et al., 2010; Geukes et al., 2013; Hajra et al., 2018). However, because these studies were mainly based on data obtained from younger adults, differences at the source-level (brain region-level), which may lead to N400 characteristic differences between younger and older adults observed at the sensor-level, remain unknown.

The current study investigates aging-related dissociation in the N400 on a semantic plausibility task at the sentence level in a verb-final language, using the ERP paradigm and source analyses. This is the first attempt to examine aging effects on semantic processing by combining the features of N400 at the sensor-level and brain activation patterns at the source-level. ERP studies on aging-related semantic processing at the sentence level have primarily focused on how aging moderates the effects of sentential context by manipulating the degree of semantic association between the preceding sentential context and the target word. Those studies have demonstrated that older adults exhibit an age-related reduction in context use compared to younger adults, which indicates that aging populations might have different levels of sensitivity to the process of contextual information. Specifically, words that were weakly associated or incongruent with preceding contextual information elicited a greater N400 effect than strongly associated or congruent words. Those semantic relatedness effects were reduced and delayed in older adults compared to younger adults (Federmeier and Kutas, 2005; Federmeier, 2007; Wlotko and Federmeier, 2012; Dave et al., 2018). Most previous evidence as reviewed above has been obtained from studies of English sentences, in which N400s were primarily driven from sentence-final nouns. However, experimental paradigms from head-initial languages, such as English, cannot be applied to verb-final languages, such as Korean, as targeted in the current study. English follows the canonical Subject–Verb–Object (SVO) word order, whereas Korean is a verb-final language with the Subject–Object–Verb (SOV) word order. Furthermore, Korean allows for the relative freedom of word order with the help of case markers as far as verbs are placed at the end of a sentence, where all semantic and syntactic integrations occurred. Case markers denote the thematic relationship of noun phrases associated with verbs. Due to these cross-linguistic differences, the semantic paradigm for sentence-level ERP studies has been modified in the current study as follows.

We manipulate semantic plausibility by varying the thematic relationship between two noun phrases (NPs) including either the theme or instrumental noun in Korean, which has case marking systems as a postpositional particle. For example, the plausible condition contains sentences such as “cut the paper (theme) with the scissors (instrumental)”, whereas the example sentence used for the implausible condition is “cut the scissors (theme) with the paper (instrumental)”. Here, in the context of being used to cut something, “scissors” is a semantically well-validated instrumental noun, while “paper” is not a semantically plausible instrumental noun. For verb-final languages, syntactic constructions were as follows: (1) Plausible: “scissors”—instrumental case marker (1st NP), “paper”—theme case marker (2nd NP), “cut” (Verb); and (2) Implausible: “paper”—instrumental case marker (1st NP), “scissors”—theme case marker (2nd NP), “cut” (Verb). Manipulating semantic plausibility by changing the instrumentality of the noun property in relation to case markers attached as postpositions is a novel approach to eliciting the N400. This paradigm was previously validated in another cross-linguistic project that employed three languages, specifically English as SVO and both Korean and Japanese as SOV languages (Sung et al., in preparation). In that study, the aging-related differences between SOV and SVO languages were investigated using a sentence-picture matching paradigm in semantic plausibility judgment tasks (plausible vs. less plausible comparisons). The current study modified the previously validated paradigms for eliciting ERP signals, replacing less plausible sentences with implausible sentences that clearly contained semantic violations. This novel semantic violation paradigm may be used in future studies investigating ERP effects in verb-final languages. We first hypothesize that the N400 effect according to the plausibility condition would be elicited, as reported in previous studies. We specifically predict that N400 effects emerge predominantly under the implausible condition which carries an odd semantic relationship between the noun property and its post-positional case marker in relation to the verbs. The results in line with the prediction would contribute to psycholinguistic validation of the semantic violation ERP paradigms in a sentence-level of a verb-final language. We further predict that the aging-related differences of the N400 effect would emerge at both the sensor- and source-levels, with different spatial and temporal resolutions, based on the assumption that aging-related neural changes may induce different brain activation patterns when processing the same semantic violation.

## Materials and Methods

### Participants

The experiment was evaluated and approved by the Institutional Review Board on Human Subjects of Ewha Womans University (2017-09-142-3). All participants gave their informed and written consent prior to experiments and the research was conducted in accordance with current guidelines and the Declaration of Helsinki.

In total 40 subjects [20 younger (age (mean ± SD) = 23.85 ± 4.37 years, range = 18–32, 16 women) and 20 older adults (age = 66.50 ± 4.48 years, range = 60–77, 16 women)] participated in the experiment. All participants were Korean native speakers with normal or corrected-to-normal vision. They were all right-handed with no reported history of neurological or psychiatric diseases and any major medical conditions based on a health screening questionnaire (Christensen et al., 1991). Participants were also screened for cognitive and neuropsychological impairments using the Korean Mini-Mental State Examination (K-MMSE; Kang et al., 2012); no one was excluded from the screening. Younger and older adults showed no significant difference in years of education (younger group = 14.80 ± 1.94 years, older group = 13.65 ± 2.23 years, *t*_(38)_ = 1.741, *p* = 0.0897).

### Materials

Experimental stimuli comprised 240 sentences in total, with 140 target sentences (70 plausible vs. 70 implausible) and 100 filler sentences. The target sentences were composed of five phrases: an instrumental post-positional phrase (PP), an accusative noun phrase (NP), an adnominal phrase (ADNP), a bound noun phrase, and a stative verb. Korean is a verb-final language with the canonical word order of SOV. In the Korean instrumental structure, arguments are canonically ordered as instrumental–accusative–verb (Im, 2007). Because the verb is placed at the end of a sentence, the effects at the verb always include both the verb effect and sentence wrap-up effect. Thus, to separate the verb effect from that of sentence wrap-up and focus on the effect derived from the verb, at which thematic information for each argument is provided and thus semantic plausibility could be determined, we nominalized the part of the instrumental structure (instrumental–accusative–verb) by transforming the sentence closing ending of the verb into the adnominal form (verb stem + the adnominal marker “*nun*”) and placing the bound noun “*kes*” subsequent to the adnominal phrase. We used an identical bound noun and stative verb for all the target sentences. The critical word was the ADNP, at which semantic plausibility is determined.

The target sentences were divided into two conditions: plausible vs. implausible. We manipulated the semantic plausibility by reversing the order of the nouns in the PP and the NP. In the plausible condition, the instrumental nouns were placed with instrumental case marker “*lo*” in the PP, followed by the theme nouns with the accusative case marker “*lul*” in the NP. In the implausible condition, the theme nouns were placed with the instrumental case marker “*lo*” in the PP, followed by the instrumental nouns with the accusative case marker “*lul*”. The semantic plausibility of the thematic relationship between the PP and the NP was determined based on the verb meaning at the ADNP. Table 1 provides sentence examples for each condition. We followed the Yale Romanization of Korean (Martin, 1992). All nouns in the PP and the NP had a frequency of more than 100, which indicates high-frequency words (Seo, 1998). Paired samples *t*-test revealed that the nouns in the PP did not significantly differ in frequency compared to those in the NP (*t*_(69)_ = −0.469, *p* = 0.6402). Considering that mental fatigue increases throughout the task to which older adults are more vulnerable (Arnau et al., 2017), we divided the 240 sentences into two sets, one of which was alternately administered to participants. Thus, each participant conducted the semantic plausibility task composed of 120 sentences including 35 plausible, 35 implausible, and 50 filler sentences.

**Table 1:** Sentence examples for the experimental conditions.

Conditions	Sentence examples
Plausible	It is possible to sharpen a pencil with a knife.
	khal-lo	yenphil-ul	kkak-nun	kes-i	kanungha-ta
	a knife-INS	a pencil-ACC	sharpen-ADN	thing-NOM	is possible-PRES-IND
Implausible	It is possible to sharpen a knife with a pencil.
	yenphil-lo	khal-ul	kkak-nun	kes-i	kanungha-ta
	a pencil-INS	a knife-ACC	sharpen-ADN	thing-NOM	is possible-PRES-IND

*The table contains sentence examples in each plausibility condition, and the critical phrases where semantic plausibility is determined are shaded. ACC, accusative; ADN, adnominal; IND, indicative mood; INS, instrumental; NOM, nominative; PRES, present*.

### Experimental Procedures

**Figure 1:**
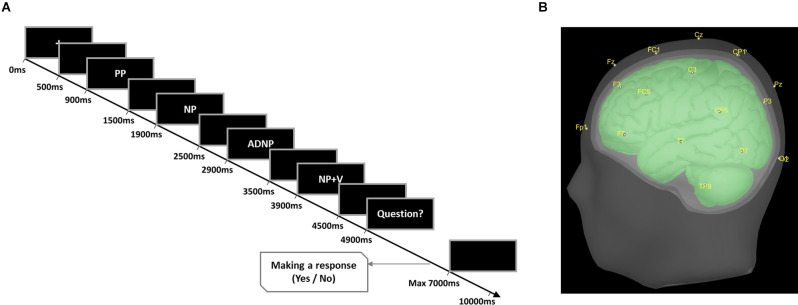
Language-related ERP experiment design. **(A)** Rapid Serial Visual Presentation paradigm. The figure shows the experimental design in which the experimental sentences are divided into four blocks and sequentially presented as visual stimuli, with the plausibility judgment task being performed at the end of the sentence. **(B)** Head model and EEG electrode placement. The figure shows the three-layer boundary element method head model reconstructed from the MNI ICBM 152 brain template, and the sensor locations on the scalp.

Participants’ EEGs were recorded in an electrically shielded room. They were seated in front of a 24-inch LCD monitor and instructed to read each sentence and judge its semantic plausibility as quickly and accurately as possible by pressing a keyboard button. To minimize artifacts on the EEG signal, they were asked to refrain from blinking or moving while sentences were presented. A practice session was administered prior to the experimental session to familiarize participants with the task. The experimental sentences were presented according to the Rapid Serial Visual Presentation paradigm (Figure 1A). Each phrase was presented serially and the last two phrases were provided together in the visual display. The stimuli were displayed in white letters on a black screen for 600 ms and at 400 ms intervals. Next, participants were asked whether the sentence was semantically plausible. The question was presented for a maximum of 7,000 ms until participants pressed a button. The inter-trial interval was 3,000 ms. The sentences were pseudo-randomly presented with the constraint of no more than three consecutive sentences belonging to the same condition. We used E-prime 2.0 software (Psychology Software Tools, Pittsburgh, PA) to display the stimulus and record behavioral responses. The experimental stimuli were divided into four blocks and participants had a short break between each. The experiment took approximately 30 min in total.

EEG data were recorded using BrainVisionBrainAmp Standard (Brain Products GmbH, Germany) with 32 actiCAP Ag-AgCl electrodes following the International 10–20 system (Fp1, Fp2, F3, F4, C3, C4, P3, P4, O1, O2, F7, F8, T7, T8, P7, P8, Fz, Cz, Pz, FC1, FC2, CP1, CP2, FC5, FC6, CP5, CP6, TP9, and TP10; Figure 1B). A single ground electrode was attached to AFz and two reference channels were attached to the right and left mastoids. The electrooculogram was recorded from an electrode below the right eye. Impedances of electrodes were kept under 10 kΩ and the sampling rate was 500 Hz.

### ERP Construction and N400 Analyses

Preprocessing of the recorded EEG data was performed based on the existing preprocessing pipeline^1^, using the EEGLAB (version 2021.0) with MATLAB (version R2019b). The continuous EEG signals were bandpass filtered at 0.1–50 Hz, and bad channels were removed and replaced through interpolation of neighboring channels (no more than one channel per participant). The signals were then re-referenced to the common average reference, and epoched at −200 to 900 ms for the critical stimuli block, ADNP (stimulus presentation time: 0 ms). After that, signal artifacts due to ocular and muscle activities or hardware system noise were removed based on Independent Component Analysis (ICA). In particular, artifact-contaminated independent components (ICs) were automatically classified using the ICLabel module (Pion-Tonachini et al., 2019), and then the contaminated ICs were finally selected and removed *via* visual inspection with reference to the classification results. Among the pre-processed segments (epochs), the segments corresponding to the stimuli that showed false responses to the plausibility question were excluded. Segments containing residual significant artifacts or noise were further identified and manually removed.

Then, only data from subjects whose remaining valid stimuli were >70% of the total stimuli (i.e., more than 24 out of 35 sentences) in both conditions were included in the subsequent analyses (*n* = 16 for younger adults and *n* = 15 for older adults). All clean segments were baseline corrected using their respective pre-stimulation intervals of −200–0 ms and ERPs were then derived by averaging the segments for all valid stimuli in each condition.

The waveforms of the constructed ERPs were examined in nine regions of interest (ROIs): left anterior (LA; F3, F7, and FC5), middle anterior (MA; Fp1, Fp2, and Fz), right anterior (RA; F4, F8, and FC6), left central (LC; T7, C3, and CP5), middle central (MC; FC1, FC2, and Cz), right central (RC; T8, C4, and CP6), left posterior (LP; P3, P7, and O1), middle posterior (MP; CP1, CP2, and Pz), and right posterior (RP; P4, P8, and O2). Because the results of previous studies (Hajra et al., 2018; Tiedt et al., 2020) and visual inspection of our data demonstrated that the N400 component was relatively clearly observed in the midline channels compared to the lateral channels (see the Results section), peak latencies were measured in MA, MC, and MP regions. These peak detections were carried out at a time window of 250–550 ms for younger adults and 300–750 ms for older adults. The reason for not using the traditional N400 time window for older adults is that delayed N400 was often observed at ~600 ms, especially in the posterior regions; furthermore, this avoids confusion with delayed N200, another component induced by aging-related effects (Elverman et al., 2021). For the amplitude analysis of N400, the mean amplitudes within the 200 ms intervals were calculated, including 100 ms before and after based on the acquired peak latencies. In the amplitude analysis, the mean amplitudes were extracted from all nine ROIs to analyze the electric potential differences in the time window in which the N400 was generated at the entire sensor-level, even though the clear N400s were not observed in all channels. In more detail, considering that peak latencies differed slightly in MA, MC, and MP, we calculated the mean amplitudes by applying the corresponding time windows according to the anteriority.

The derived individual peak latencies and mean amplitudes were statistically compared *via* three-way mixed ANOVA with a group (younger and older adults), a plausibility condition (plausible and implausible), and a channel region (MA, MC, and MP for the peak latency, and nine ROIs for the mean amplitude) as independent variables. In both analyses, the between-subjects factor was set as the group and the within-subject factors were set as the plausibility condition and channel region. The significance level was defined as *p* < 0.05 and *post-hoc* analyses were performed for significant results using paired samples t-test or independent-samples t-test with Bonferroni correction. If the assumption of sphericity was violated, Greenhouse-Geisser correction was applied (Greenhouse and Geisser, 1959). For the statistical analyses, IBM SPSS STATISTICS version 22 was employed. The individual topographic patterns for the N400 were further analyzed using the electric potentials averaged within individual time windows of 200 ms, defined based on the N400 peak latencies in the MP region where both the N400s and condition-dependent N400 effects were clearly observed (see the Results section).

### ERP Source Estimation and N400 Source Analyses

Source activities in the brain (cerebral cortex) were estimated from the constructed ERPs of individual participants, using Brainstorm (Tadel et al., 2011). A three-layer boundary element method (BEM) head model was constructed from the MNI ICBM 152 brain template (Fonov et al., 2009, 2011), with the following relative conductivities: scalp = 1.0, skull = 0.0125, and brain = 1.0 (Mahjoory et al., 2017). In the source space, dipoles were placed at each vertex of the triangulated surface mesh with orientations perpendicular to the surface at each vertex of the cerebral cortex. The total number of dipoles (source positions) was 15,002, and the average distance between them was ~4.8 mm. Regarding the EEG sensor placement, the standard coordinate information of actiCAP 32 channels, included as an option in Brainstrom, was employed. Because structural brain images and electrode positions of individual participants were not available here, the head model and sensor placement constructed above were applied to all participants (Figure 1B).

Forward modeling to generate the lead field matrix for source-to-sensor projection was performed *via* the symmetric BEM implemented in OpenMEEG (Gramfort et al., 2010). Next, inverse modeling to estimate source activities in the brain was conducted using the obtained forward model and averaged ERPs (by condition) of individual participants. Noise covariances were calculated from pre-stimulus recordings (−200–0 ms) of individual trials for each subject, and current densities at 15,002 source locations were then computed for every timepoint of the epoch by applying minimum norm estimation with dynamical statistical parametric mapping (dSPM) normalization (Dale et al., 2000; Tadel et al., 2011, 2019; Stropahl et al., 2018). As a result, for each ERP, the time series of source activities were acquired with the same interval as the epoch (−200 to 900 ms).

Prior to extracting source activation patterns in N400 time windows, spatial smoothing was applied to the estimated source signals of individual subjects, using a Gaussian kernel with a full width half maximum of 3 mm (Tadel et al., 2019). To interpret the estimated current densities in terms of brain activation regardless of polarity, we used absolute current strengths in the analyses (Tadel et al., 2019). The current strengths were then averaged over the individual N400 time windows defined above so that for each subject, N400 source activation patterns for each plausibility condition were derived. Differences in N400 source activation patterns according to plausibility conditions were identified in each group by paired samples t-test using the obtained values at each source (vertex) location (significance level: *p* < 0.05). The Desikan-Killiany cortical atlas was used to identify regions showing significant differences according to plausibility conditions (Desikan et al., 2006).

Through vertex-wise analyses, regions that revealed marked differences in source activities according to the plausibility conditions in each group were defined as ROIs (significance level: *p* < 0.01), and further statistical analyses were performed using the averaged current strengths in the corresponding ROIs. The ROI-wise source activities were compared *via* three-way mixed ANOVA with the group, plausibility condition, and source region as independent variables, as in sensor-level analyses (between-subjects factor = group, within-subject factors = plausibility condition and source region). The significance level was defined as *p* < 0.05, and the corrected significance thresholds based on Bonferroni correction were used for *post-hoc* analyses.

## Results

Sixteen younger adults and 15 older adults who met the criteria for the number of remaining trials (>70% of the total stimuli, for each condition)—excluding trials with false responses or severe signal artifacts—were included in the analyses (Table 2). The included younger and older adults showed no significant differences in years of education (*t*_(29)_ = 1.562, *p* = 0.1291) and K-MMSE scores (*t*_(20.2)_ = 2.051, *p* = 0.0534). During EEG preprocessing, the included subjects did not show significant differences between groups in terms of the number of bad channel interpolations (*t*_(29)_ = −1.518, *p* = 0.1400) and the number of ICA-based artifact removals (*t*_(29)_ = −0.749, *p* = 0.4597). However, there was a significant difference between the two groups in terms of the number of trials included in the analyses (*t*_(29)_ = 2.050, *p* = 0.0495).

**Table 2:** Characteristics of participants included in the final analyses.

	Younger group (*n* =16)	Older group (*n* =15)
Demographic data
Age*	24.38 ± 3.88 (range = 18–32)	67.00 ± 5.04 (range = 60–77)
Years of education	15.13 ± 1.89	13.93 ± 2.34
K-MMSE	29.75 ± 0.58	29.07 ± 1.16
EEG preprocessing
Bad channel interpolation	none	0.13 ± 0.35
ICA-based artifact removal	3.19 ± 1.56	3.60 ± 1.50
The number of trials included in analyses*	60.13 ± 4.81	55.93 ± 6.50
Task performance (the number of sentences with true responses)
Plausible condition	32.75 ± 1.73	31.47 ± 2.62
Implausible condition	33.19 ± 2.66	32.67 ± 1.80

*Asterisks indicate variables with significant group differences*.

### Behavioral Analyses

Two-way mixed ANOVA (group * plausibility condition: plausible, implausible) revealed that none of the comparisons were significant: the main effects for the group (*F*_(1,29)_ = 2.015, *p* = 0.1664, ɛ^2^ = 0.020) and the condition (*F*_(1,29)_ = 2.708, *p* = 0.1107, ɛ^2^ = 0.021), and the interaction between them (*F*_(1,29)_ = 0.587, *p* = 0.4497, ɛ^2^ = −0.005). Table 2 presents the task performance by condition in each group.

### N400 ERP Analyses

#### Peak Latency

**Figure 2:**
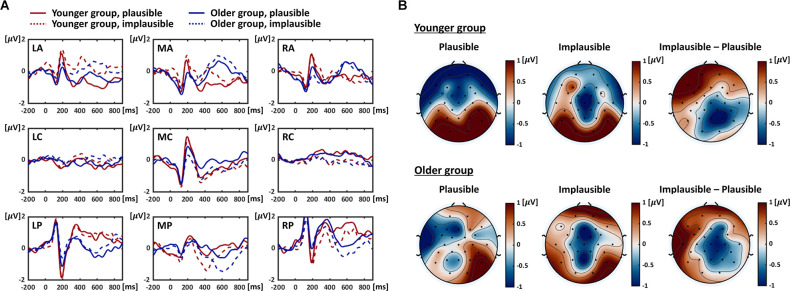
Grand-averaged ERPs and N400 topographic patterns. **(A)** Grand-averaged ERPs according to plausibility conditions in two age groups. The figures show the averaged ERP signals obtained from nine channel groups. The red and blue lines represent the signals acquired in the younger and older groups, and the solid and dotted lines indicate the signals derived from the plausible and implausible condition respectively. LA, left anterior; LC, left central; LP, left posterior; MA, middle anterior; MC, middle central; MP, middle posterior; RA, right anterior; RC, right central; RP, right posterior. **(B)** N400 topographic patterns in two age groups. The figures present the averaged N400 topographic patterns derived based on individual time windows for the N400. In the sub-figures for each group, the left and middle columns show the results for each plausibility condition, and the right columns exhibit the differences between them (implausible–plausible). The color code indicates the scalp voltage on each channel.

Three-way mixed ANOVA (group * plausibility condition * channel region: MA, MC, MP) demonstrated a significant main effect for the group (*F*_(1,29)_ = 43.147, *p* < 0.0001, ɛ^2^ = 0.352), with longer latency in the older group (younger group = 391.5 ± 15.7ms, older group = 539.8 ± 16.2 ms); it also yielded a significant main effect for channel region (*F*_(2,58)_ = 8.537, *p* = 0.0006, ɛ^2^ = 0.054), and the *post-hoc* analysis revealed that N400 latency in the MP region was longer than in the MA and MC regions (MA = 444.4 ± 18.8ms, MC = 443.9 ± 16.5 ms, MP = 508.7 ± 9.1 ms; *p* = 0.0108 for MP > MA, *p* = 0.0024 for MP > MC). Neither the main effect for the condition nor the interactions between each factor were significant. Grand-averaged ERPs show the preceding findings at a glance (Figure 2A). Visual inspection from the grand-averaged ERPs suggests that the older group clearly demonstrated the delayed N400, particularly in the MP region across conditions, whereas the younger group did not show considerable differences in N400 latency by the channel region.

#### Mean Amplitude

Repeating the three-way mixed ANOVA (group * plausibility condition * channel region: nine ROIs) demonstrated a significant main effect for the channel region (*F*_(3.4,98.3)_ = 4.086, *p* = 0.0066, ɛ^2^ = 0.070). The *post-hoc* analysis revealed that there were no pairs of channel regions with significant differences in mean amplitude (corrected significance threshold = 0.0013); however, it derived relatively low *p values* between LP and MP (*p* = 0.0083) and between MP and RP (*p* = 0.0055), respectively (LP = 0.377 ± 0.198 μV, MP = −0.826 ± 0.202 μV, RP = 0.463 ± 0.267 μV). The interaction between condition and channel region was also significant (*F*_(4.3, 125.1)_ = 7.674, *p* < 0.0001, ɛ^2^ = 0.030), and *post-hoc* analysis indicated that the implausible condition generated the N400 with greater amplitude (more negative) than the plausible condition in MP (plausible = −0.442 ± 0.248 μV, implausible = −1.202 ± 0.205 μV, *p* = 0.0016), RC (plausible = 0.482 ± 0.192 μV, implausible = −0.004 ± 0.201 μV, *p* = 0.0006) and RP regions (plausible = 0.819 ± 0.286 μV, implausible = 0.134 ± 0.293 μV, *p* = 0.0015), and the N400 with the smaller amplitude in the LA region (plausible = −0.691 ± 0.289 μV, implausible = 0.201 ± 0.277 μV, *p* = 0.0001). The ANOVA showed no significance for the main effects for the group and condition, or for other interactions. Figure 2A exhibits the grand-averaged ERPs by plausibility condition in each group, visualizing the former result, i.e., N400 effect identified mainly in the central and posterior regions of the middle and right sides.

#### Topographic Pattern

For each individual subject, the EEG topographic pattern for the N400 was obtained from the averaged potentials in each channel within the individual time windows, which are defined based on the peak latencies in the MP region where the N400 peak and the N400 effect according to the plausibility conditions were clearly observed. The averaged N400 topographic patterns in the younger group demonstrated that stronger negative potentials occurred in the central-posterior regions under the implausible condition compared to the plausible condition and those negative potentials were biased toward the right side (Figure 2B). In the older group, the differences in the N400 topographic patterns according to the plausibility conditions (implausible–plausible) were generally similar to those in the younger group but were the most pronounced in the middle-central regions (Figure 2B).

### N400 Source Analyses

**Figure 3:**
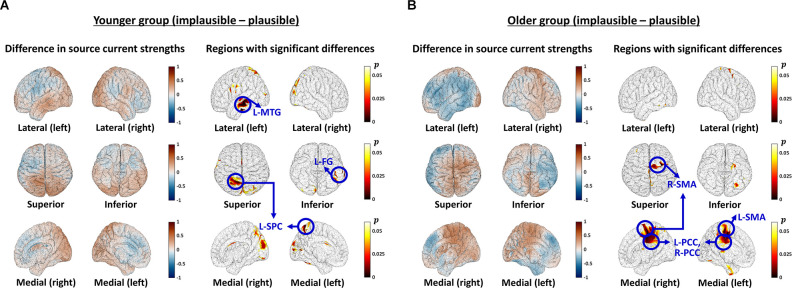
Differences in brain activation patterns according to plausibility conditions. **(A,B)** Outcomes in the younger **(A)** and older **(B)** groups. In each figure, the left columns present the average results across the whole-brain, indicating brain areas that are more (red) or less (blue) activated in the implausible condition than in the plausible condition. The right columns exhibit the areas with significant differences depending on the plausibility conditions, and the color code indicates *p* values. The areas that showed distinct differences with significance level *p* < 0.01 are marked by blue circles. The sub-figure depicts the results in the lateral (left/right) view, superior/inferior view, and medial view (left/right). L-MTG, left middle temporal gyrus, L-FG, left fusiform gyrus, L-SPC, left superior parietal cortex, L-(R-)SMA, left (right) supplementary motor area, L-(R-)PCC, left (right) posterior cingulate cortex.

The brain activation patterns of the N400 were defined as the average values of the source-estimated current densities (strengths at each brain node) within individual time windows for each subject. When comparing the activation patterns in the younger group, five regions were found to be more involved in the implausible condition than the plausible condition (*p* < 0.05): (1) the left temporal cortex [mainly the middle temporal gyrus (MTG)]; (2) the left fusiform gyrus (FG); (3) the left superior parietal cortex (SPC); (4) the bilateral precuneus and cuneus cortices; and (5) the bilateral lateral occipital cortices [mainly the parts adjacent to (1) and the parieto-occipital region] (Figure 3A). Conversely, the left pars opercularis and the inferior part of the left postcentral gyrus were observed as less engaged in the implausible condition.

In the older group, regions including: (1) the right superior frontal gyrus (mainly the posterior part adjacent to the precentral gyrus); (2) the right caudal middle frontal gyrus; (3) the bilateral paracentral lobules; (4) the bilateral posterior cingulate cortices (PCCs); and (5) the right pars opercularis (the part of sulcus adjacent to the rostral middle frontal gyrus) were identified as more involved in the implausible condition than in the plausible condition (*p* < 0.05; Figure 3B). In contrast, the left FG showed lower source activity under the implausible condition.

Based on the above vertex-wise analyses, the regions that indicated marked differences (*p* < 0.01) between the plausibility conditions in each group were selected as ROIs, and further statistical analyses were conducted. The posterior part of the superior frontal gyrus, the caudal middle frontal gyrus, and the paracentral lobule identified in the older group were grouped as one ROI, the supplementary motor area (SMA). Therefore, a total of seven ROIs were employed in the analyses (Figure 3): the left MTG (L-MTG), the left FG (L-FG), the left SPC (L-SPC), the left/right SMAs (L-SMA and R-SMA), and the left/right PCCs (L-PCC and R-PCC). Detailed information on ROI locations is provided in Supplementary Figure S1.

Three-way mixed ANOVA (group * plausibility condition * source region: seven ROIs) demonstrated a significant main effect for the plausibility condition (*F*_(1,29)_ = 5.491, *p* = 0.0261, ɛ^2^ = 0.026), with greater source activity under the implausible condition (plausible = 0.741 ± 0.044, implausible = 0.873 ± 0.049). The three-way interaction (group * plausibility condition * source region) was also significant (*F*_(2.9,83.5)_ = 11.142, *p* < 0.0001, ɛ^2^ = 0.048), and *post-hoc* analysis within each group revealed that the interactions between condition and source region were significant in both groups (younger group = (*F*_(2.8,42.7)_ = 4.025, *p* = 0.0144, ɛ^2^ = 0.029), older group = (*F*_(2.5,34.8)_ = 7.970, *p* = 0.0007, ɛ^2^ = 0.067). Table 3 presents the results of subsequent *post-hoc* analyses comparing the effect of the plausibility condition for each source region. Considering the corrected significance threshold, 0.0071, L-MTG in the younger group and R-SMA in the older group were found to generate larger source activities under the implausible condition compared to the plausible condition.

**Table 3:** Comparison of source activity according to plausibility condition for each ROI (within each group).

ROIs	Younger group - Source current density (strength)				Older group - Source current density (strength)			
	Plausible	Implausible	*p* value	Effect size	Plausible	Implausible	*p* value	Effect size
L-MTG	0.668 ± 0.057	0.939 ± 0.095	0.0035*	0.764	0.897 ± 0.145	0.731 ± 0.068	0.2505	0.271
L-FG	0.668 ± 0.064	0.881 ± 0.082	0.0173	0.592	0.867 ± 0.105	0.715 ± 0.084	0.2385	0.279
L-SPC	0.729 ± 0.066	1.008 ± 0.103	0.0153	0.605	0.872 ± 0.085	0.990 ± 0.117	0.4632	0.171
L-SMA	0.784 ± 0.080	0.776 ± 0.087	0.9306	0.020	0.660 ± 0.065	1.001 ± 0.096	0.0124	0.649
R-SMA	0.820 ± 0.079	0.853 ± 0.111	0.7560	0.070	0.697 ± 0.061	1.047 ± 0.101	0.0067*	0.719
L-PCC	0.727 ± 0.092	0.695 ± 0.085	0.7320	0.077	0.618 ± 0.070	0.934 ± 0.118	0.0084	0.693
R-PCC	0.735 ± 0.091	0.695 ± 0.083	0.6670	0.097	0.627 ± 0.081	0.955 ± 0.119	0.0082	0.696

*Asterisks indicate significant differences. L-MTG, left middle temporal gyrus; L-FG, left fusiform gyrus; L-SPC, left superior parietal cortex; L-(R-)SMA, left (right) supplementary motor area; L-(R-)PCC, left (right) posterior cingulate cortex. The effect size was presented based on the corrected Cohen’s D (Durlak, 2009)*.

## Discussion

The current study investigated aging-related differences in sentence processing containing semantic plausibility manipulations, by employing an ERP paradigm. We manipulated the semantic plausibility by varying the noun property that is marked by the instrumental case markers. By focusing on the N400 components of the acquired ERPs, one of the biomarkers of semantic processing (Hajra et al., 2018), we systematically explored differences between younger and older groups at the EEG sensor-level and brain regional source-level. An overview of the current results showed that significant differences emerged between younger and older groups in the peak latency of N400 and its related source locations, whereas there were no significant group differences in accuracy from the behavioral analyses and in the mean amplitude of N400. It is interesting to note that the accuracy and mean amplitude of N400 turned out to be the outcome variables that did not contribute to differentiating an aging population from the younger group, while the peak latency and the source activation pattern of N400 were identified as the critical factors that elicited aging-related dissociations in the current semantic plausibility paradigm.

In terms of peak latency of the N400, significant group differences were identified from the statistical analysis results, indicating that the peak latency was longer in the older group. These findings are consistent with previous results that reported a clear delay of the N400 in the older group (Kutas and Iragui, 1998; Federmeier and Kutas, 2005; Wlotko et al., 2010). The researchers speculated that the delayed N400 reflects an aging-related decline in processing speed and a reduction in working memory capacity for both information retrieval and integration processes (Salthouse, 1992, 1996; Federmeier and Kutas, 2005; Wlotko et al., 2010). The main effect for the channel region was also significant, indicating longer latency in the posterior region than in the anterior and central regions. Although the interaction between the group and channel region was not statistically significant (*p* = 0.0620), visual inspection of the grand-averaged ERPs suggests that the characteristics in the older group with a clear delayed N400 in the posterior region may have derived the result. Meanwhile, none of the effects related to the plausibility condition were significant, demonstrating that the delayed feature of N400 in the older group was universally observed. The results may indicate that the peak latency reflects a general slowdown of cognitive processing associated with aging across plausibility manipulations. As we confirmed the delayed peak latency in the older group, we observed a wide range of individual variability for the time window where the N400 component was observed. To take this into account, we individually defined N400 time windows based on peak latencies and employed them for the following analyses.

With respect to the mean amplitude of the N400, the statistical analysis results demonstrated that the main effect of the channel region was significant. Even though *post-hoc* analysis did not extract the channel region pairs representing significant differences in the mean amplitude among the nine ROIs, relatively low *p* values were identified between LP and MP regions (*p* = 0.0083) and between MP and RP regions (*p* = 0.0055). These results were derived from that the MP region had a lower amplitude than the LP and RP regions, which is consistent with previous studies in which the N400 component was generally observed in the centro-parietal regions (Hajra et al., 2018; Payne and Federmeier, 2018; Tiedt et al., 2020). The effect of the channel region further significantly interacted with the plausibility condition, revealing that the condition-dependent N400 effect (i.e., the larger negative peak under the implausible condition) was mainly induced in the central and posterior regions across the groups. These results are consistent with previous findings that reported that the semantically implausible conditions elicited a larger N400 (Kutas et al., 2007; Kutas and Federmeier, 2011; Hajra et al., 2018; Payne and Federmeier, 2018; Tiedt et al., 2020). The researchers have interpreted the greater N400 as an index to represent greater cognitive demand for resolving semantic anomalies (Kutas et al., 2007; Kutas and Federmeier, 2011). Meanwhile, our study did not yield any statistically significant results in terms of group differences in N400 amplitude. These results are not aligned with those of several previous studies in which the condition-dependent N400 effect was significantly attenuated in older adults (Federmeier and Kutas, 2005; Federmeier et al., 2010; Payne and Federmeier, 2017, 2018; Tiedt et al., 2020). This can likely be attributed to the differences in the characteristics of experimental stimuli. In the current study, we presented a context consisting of two phrases only, including nouns and case markers, and analyzed the N400 at the verb. In contrast, previous studies of English sentences mainly had presented a full sentence or more than a full sentence as context to analyze the N400 at the sentence-final noun. Considering the context input, the current study used relatively shorter sentences, which may have reduced processing demands, leading to reduced age-related differences in the N400 amplitude at the target word. This speculation of a relatively reduced processing demand imposed by the current paradigm is also supported by our behavioral results, where older adults did not significantly differ from the younger group in the semantic plausibility judgment.

Although statistical analysis for the mean amplitude of N400 did not yield significant results for group differences, the topographic patterns of the N400 in the EEG sensor space demonstrated an interesting dissociation between the two groups: in the younger group, the condition-dependent N400 effect was mainly observed in the right-biased central-posterior regions, and in the older group, it was identified around the middle central regions. To scrutinize how those group differences were associated with neural activation patterns at the brain region-level, we further performed source analyses on the N400.

The source estimation results demonstrated that different regions were recruited depending on the plausibility conditions in each group. In the younger group, the left MTG was found to be most clearly engaged in the implausible condition rather than the plausible condition, and the left FG and left SPC were also found to be more involved in the implausible condition with medium to large effect sizes. Given that the current experiment employed the semantic violation paradigm, the results are clearly aligned with previous findings that suggest the left temporal region is the largest source for the N400 effect associated with semantic context (Van Petten and Luka, 2006; Khateb et al., 2010; Geukes et al., 2013; Hajra et al., 2018). Furthermore, the left MTG and left FG that were recruited more under the implausible condition are core areas belonging to the general lexical-semantic networks as reported in previous studies, especially based on functional MRI (Binder et al., 2009; Binder and Fernandino, 2015; Liuzzi et al., 2020).

In contrast, in the older group, the bilateral regions spanning from the posterior regions of the superior frontal gyrus (adjacent to the precentral gyrus) to the medial areas, including the paracentral lobules and PCCs, were found to be more involved under the implausible condition; these patterns were more pronounced in the right-hemisphere than in the left-hemisphere, and in the left-hemisphere, they were mainly observed in the medial areas. These regions considerably overlap with the SMAs that have been reported as core areas for multiple-demand networks (Müller et al., 2015; Davey et al., 2016; Hoffman and Morcom, 2018). In particular, a review on the functions of SMA suggested that the SMA carries out diverse superordinate control functions for language perception and processing, especially when task demands increase (Hertrich et al., 2016). When considering that the implausible condition contains semantic violations, which may impose greater cognitive-linguistic processing demands than the plausible condition, the source analysis results suggest that processing implausible sentences recruits more cognitive resources. It is interesting to note that we found aging-related differences in this regard. Younger adults seemed to rely on the brain regions associated with lexical-semantic functions when resolving semantic violations, whereas older adults seemed to induce more engagement of the brain regions related to controlled attention or superordinate executive functions.

Another interesting difference in group comparisons was right-hemisphere involvement. The source estimation results demonstrated that older adults activated right-hemisphere regions with medium to large effect sizes, which contrasted with younger adults, who engaged the left-lateralized regions. These findings are consistent with previous studies that reported that attenuated activation in the left-hemisphere regions and enhanced activation in the right-hemisphere regions were identified in older adults with respect to control and regulation of semantic processing (Cabeza, 2002; Hoffman and Morcom, 2018). Recruitment of the nondominant hemisphere is generally known to be associated with inefficient or more dedifferentiated neural processing (Morcom and Johnson, 2015; Knights et al., 2021) and to reflect compensatory mechanisms that may contribute to preserving linguistic and cognitive function, especially among older adults (Wingfield and Grossman, 2006; Bergerbest et al., 2009; Cabeza et al., 2018) or in patients with function degradation due to brain damage (Thiel et al., 2001; Riès et al., 2016). Given that the older adults in this study did not show any significant difference from the younger adults in the plausibility judgment, our source analysis results may suggest that older adults could achieve similar task performance to younger adults, by compensating for the under-activation of the left-lateralized core semantic network through the recruitment of the bilateral or right multiple-demand network. In other words, the results demonstrated that older adults rely on more domain-general processing resources to resolve semantic violations, which may support age-related compensation mechanisms based on neurocognitive flexibility (Lövdén et al., 2010; Hoffman and Morcom, 2018).

Meanwhile, further ROI-based statistical analyses more clearly revealed the results for source activities described in the above paragraphs. First, the main effect for the plausibility condition was found to be significant, showing that larger source activities were generated under the implausible condition than the plausible condition, across the groups. The result indicates that more cognitive resources were devoted to processing sentences with semantic violations in both groups. The statistical analyses further identified that the three-way interaction between each factor was significant, and subsequent analyses demonstrated that the two groups activated distinct brain regions when resolving the same semantic violations. In particular, it was confirmed that the younger group actively involved the L-MTG, which is a core region of the lexical-semantic network, whereas the older group recruited SMAs (mainly in the right hemisphere), which are responsible for superordinate control functions, as parts of the multiple-demand network.

To summarize, the current study examined aging-related differences in sentence processing using the semantic violation plausibility judgment paradigm in a verb-final language. Both younger and older groups seemed to recruit more cognitive resources under the implausible condition to resolve semantic conflicts. In contrast, age-related dissociations clearly emerged in the functional networks that each group recruited for resolving the semantic conflicts. To the best of our knowledge, the current study is the first attempt to determine age-group differences through EEG source analyses under the semantic violation paradigm in a sentential level for those who use a verb-final language. Despite a relatively small sample size, it is noteworthy that we found significant group differences with fairly good effect sizes in the peak latency and its related source analyses. In addition to the extension of sample size with language diversities, we suggest that future studies need to further elaborate the approaches of source analyses. In order to identify the differences in the source locations between the two plausibility conditions, the current study analyzed the magnitude differences based on the source current strengths as used in several previous studies (Geukes et al., 2013; Sokoliuk et al., 2019). We presented the results on general N400-related brain activation regardless of the polarity of the source current. However, this approach makes it difficult to explore the underlying mechanisms involving the polarity of the current and to scrutinize how the direction of the current in each brain region is associated with behavioral results. Further studies are required to examine the aging-related group differences at a sentence-level by reflecting the polarity of the source current in larger samples.

The current study delivers some critical implications in that it provides an evidence-based experimental paradigm for researchers who endeavor to elaborate aging studies using ERP and source analyses in a verb-final language. Further studies are required to conduct more in-depth analyses of individual variables such as neuropsychological test scores and demographic characteristics as potentially mediating factors that account for age-related differential patterns of activated brain networks. From the perspective of utilizing multiple EEG characteristics, we suggest that researchers consider employing the power spectral density and functional connectivity (Ewald et al., 2012; Teng et al., 2018; Packard et al., 2020) in addition to the specific component of ERP that we focused on in this study to explore the underlying cognitive mechanisms associated with the aged brain in more detail. Despite some limitations of the study, it has great importance in that it demonstrated that our task paradigm is effective at revealing the aging effects on sentence processing in ERP peak latency and its related brain source identification under the sentential-level semantic violation paradigm in a verb-final language.

## Data Availability Statement

The original contributions presented in the study are included in the article/Supplementary Material, and further inquiries can be directed to the corresponding author/s.

## Ethics Statement

The studies involving human participants were reviewed and approved by Institutional Review Board on Human Subjects of Ewha Womans University (2017-09-142-3). The patients/participants provided their written informed consent to participate in this study.

## Author Contributions

SA contributed to analysis and interpretation of the data and wrote the main manuscript text. SJO contributed to data acquisition, interpretation of the data, and wrote the main manuscript text. SBJ contributed to interpretation of the data and substantively revised the article. JES contributed to the conception of the work, interpretation of the data, and substantively revised the article. All authors contributed to the article and approved the submitted version.

## Conflict of Interest

The authors declare that the research was conducted in the absence of any commercial or financial relationships that could be construed as a potential conflict of interest.

## Publisher’s Note

All claims expressed in this article are solely those of the authors and do not necessarily represent those of their affiliated organizations, or those of the publisher, the editors and the reviewers. Any product that may be evaluated in this article, or claim that may be made by its manufacturer, is not guaranteed or endorsed by the publisher.

## Footnotes

^1^ https://sccn.ucsd.edu/wiki/Makoto’s_preprocessing_pipeline
