# Supplementary material for: Aging-Related Dissociation of Spatial and Temporal N400 in Sentence-Level Semantic Processing: Evidence From Source Analyses
Source: Front Aging Neurosci. 2022 Jun 10;14:877235. doi: 10.3389/fnagi.2022.877235 (PMC9226558; doi:10.3389/fnagi.2022.877235)
Supplement: Supplementary file 1 [file Data_Sheet_1.DOCX]

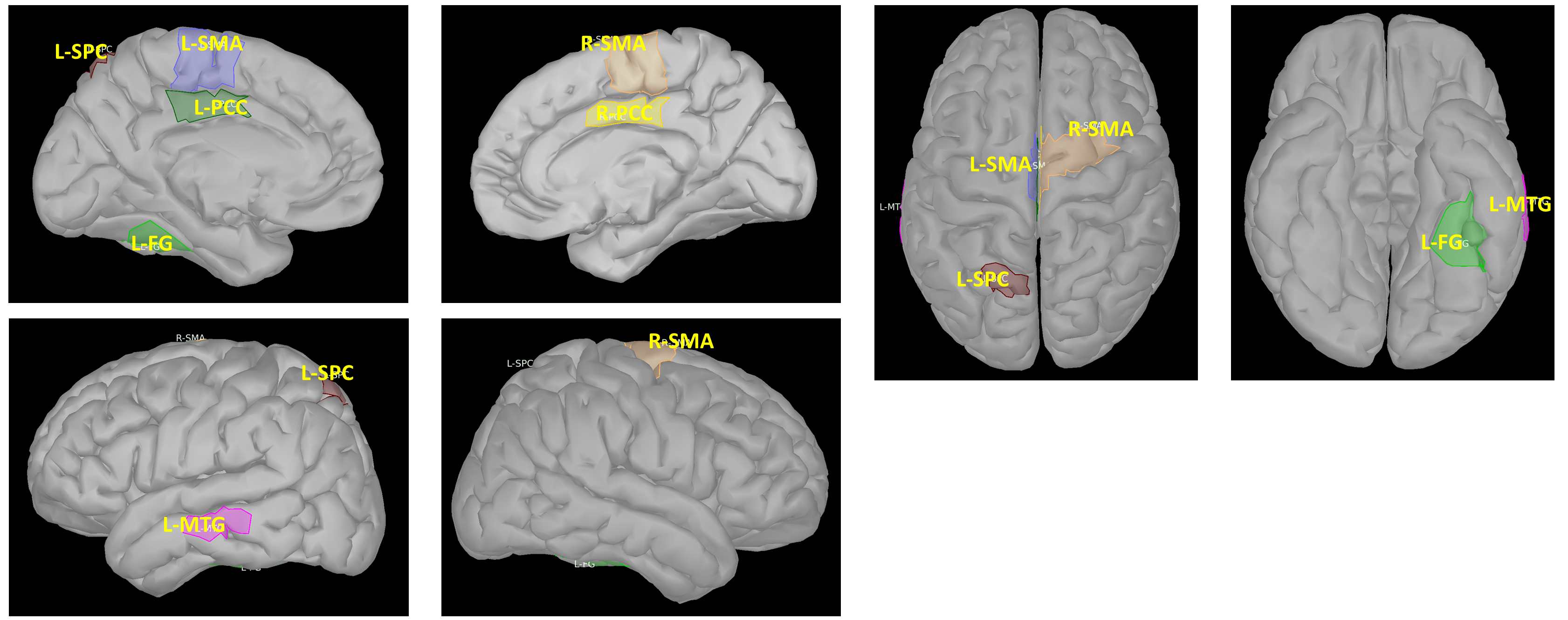


Figure S1. Seven ROIs employed for source analyses. The figures show the locations of seven ROIs used for comparison of source activity according to plausibility condition. L-MTG = left middle temporal gyrus, L-FG = left fusiform gyrus, L-SPC = left superior parietal cortex, L-(R-)SMA = left (right) supplementary motor area, L-(R-)PCC = left (right) posterior cingulate cortex.
